# The Impact of COVID-19 on Forensic Rehabilitation in Austria

**DOI:** 10.1192/j.eurpsy.2023.485

**Published:** 2023-07-19

**Authors:** M. Koch, A. Dvorak, M. Hobersdorfer, L. Yeghiazaryan, U. Rabl, A. Komorowski

**Affiliations:** ^1^Medical University of Vienna, Vienna; ^2^Justizanstalt Göllersdorf, Göllersdorf, Austria

## Abstract

**Introduction:**

In general, forensic psychiatric patients experience major restrictions of freedom. To mitigate the risk for the coronavirus disease 2019 (COVID-19), even more restrictions were implemented in Austrian mental health institutions in 2020. Besides potential deterioration of psychopathological symptoms, exaggerated freedom-restricting measures may limit the forensic rehabilitation of offenders.

**Objectives:**

Given that rehabilitative efforts and social visits were suspended for more than a year, this study aimed to evaluate the impact of the COVID-19 pandemic on the psychosocial rehabilitation of forensic psychiatric patients.

**Methods:**

This retrospective longitudinal observational study evaluated institutional data before and after the enactment of freedom-restricting measures in an Austrian forensic mental health institution. Data were obtained from 97 offenders treated at the institution *Justizanstalt Göllersdorf* during two time periods (January 2019 – March 2020 and March 2020 – May 2021). Statistical differences between both periods were assessed by means of Wilcoxon signed-rank tests. Study outcomes included the number of visits by relatives and legal guardians as well as rehabilitative activities.

**Results:**

After the outbreak of the pandemic, access to penal institutions was limited for external visitors, which led to a decrease in visits by relatives (1440 vs. 394, p < .001) and legal guardians (286 vs. 122, p = .003). Further, the total number of one-day temporary releases of patients (64 vs. 3, p < .001) and group excursions (103 vs. 10, p < .001) decreased in the second study period.

**Conclusions:**

Focusing on social contacts and rehabilitative activities, this study highlights the impact of the current pandemic in forensic psychiatry. While COVID-19-related protective measures may reduce the risk for disease transmission, enforced quarantine and other restrictions of freedom impair the rehabilitation of forensic psychiatric patients. This implies the necessity for guidelines to uphold an appropriate standard of care during future pandemics.

**Disclosure of Interest:**

None Declared

